# Abnormal Neurodevelopmental Outcomes are Common in Children with Transient Congenital Hyperinsulinism

**DOI:** 10.3389/fendo.2013.00060

**Published:** 2013-05-20

**Authors:** Hima Bindu Avatapalle, Indraneel Banerjee, Sajni Shah, Megan Pryce, Jacqueline Nicholson, Lindsey Rigby, Louise Caine, Mohammed Didi, Mars Skae, Sarah Ehtisham, Leena Patel, Raja Padidela, Karen E. Cosgrove, Mark J. Dunne, Peter E. Clayton

**Affiliations:** ^1^Department of Paediatric Endocrinology, Royal Manchester Children’s HospitalManchester, UK; ^2^Manchester Academic Health Sciences Centre, Faculty of Medical and Human Sciences, Royal Manchester Children’s Hospital, University of ManchesterManchester, UK; ^3^Department of Clinical Psychology, Royal Manchester Children’s HospitalManchester, UK; ^4^Department of Paediatric Endocrinology, Alder Hey Children’s HospitalLiverpool, UK; ^5^Faculty of Life Sciences, University of ManchesterManchester, UK

**Keywords:** congenital hyperinsulinism, hypoglycemia, neurodevelopment, developmental delay, neurological outcome, seizures, transient congenital hyperinsulinism

## Abstract

**Introduction:** Neuroglycopenia is recognized to be associated with abnormal neurodevelopmental outcomes in 26–44% of children with persistent congenital hyperinsulinism (P-CHI). The prevalence of abnormal neurodevelopment in transient CHI (T-CHI) is not known. We have aimed to investigate abnormal neurodevelopment and associated factors in T-CHI and P-CHI.

**Materials and Methods:** A cohort of children with CHI (*n* = 67, age 2.5–5 years) was assessed at follow-up review and noted to have normal or abnormal (mild or severe) neurodevelopmental outcomes for the domains of speech and language, motor, and vision. Children were classified as P-CHI (*n* = 33), if they had undergone surgery or remained on medical therapy, or T-CHI (*n* = 34), if medical treatment for hypoglycemia was stopped.

**Results:** Overall, abnormal neurodevelopment was present in 26 (39%) children with CHI, of whom 18 (69%) were severe. Importantly, the incidence of abnormal neurodevelopment in T-CHI was similar to that in P-CHI (30 vs. 47% respectively, *p* = 0.16). The prevalence of severe abnormal neurodevelopment in speech, motor, and vision domains was similar in both T-CHI and P-CHI children. For this cohort, we found that the severity of disease [based upon maximal diazoxide dose (odds ratio 95% confidence intervals) 1.3 (1.1; 1.5), *p* = 0.03], and early presentation of CHI <7 days following birth [5.9 (1.3; 27.8), *p* = 0.02] were significantly associated with abnormal neurodevelopment. There was no significant association with gender, genotype, or the histopathological basis of CHI.

**Conclusion:** Abnormal neurodevelopment was evident in one third of children with both T-CHI and P-CHI, early presentation and severe CHI being risk factors. Early recognition and rapid correction of hypoglycemia are advocated to avoid abnormal neurodevelopment in children with CHI.

## Background

Congenital Hyperinsulinism (CHI) is caused by dysregulated excessive insulin secretion leading to profound and unpredictable hypoglycemia, often in early childhood. Severe hypoglycemia in CHI can lead to neuroglycopenia in the brain, associated with suppression of other metabolites such as ketones or lactate, which usually preserve neuronal function in the absence of glucose (Arnoux et al., 2012; Senniappan et al., 2012). However, the association of developmental delay and epilepsy in children with diabetes with DEND syndrome (developmental delay, epilepsy, and neonatal diabetes) also raises the possibility that gene defects common to the pancreas and the CNS can contribute to altered neurological function (Gloyn et al., 2006). Abnormal neurodevelopmental outcomes with a tendency to motor and speech delay in early childhood have been recognized in children with persistent CHI (P-CHI) (Arnoux et al., 2012) with incidence varying between 26 and 44% in relatively large and well characterized cohorts of patients (Menni et al., 2001; Meissner et al., 2003; Ludwig et al., 2011). The prevalence of abnormal neurodevelopment and seizures appears to be particularly high in CHI associated with hyperammonemia (Bahi-Buisson et al., 2008; Kapoor et al., 2009), while infantile spasms, a rare seizure disorder, has also been described in children with P-CHI (Kumaran et al., 2010). In contrast, a significant proportion (up to 50%) of children with CHI, achieve spontaneous resolution without a long-term need for medication and/or surgery; transient CHI (T-CHI) (Banerjee et al., 2011). Yet, only one single case report has described T-CHI with unfavorable neurological outcome (Vercellino et al., 2011), and there are no reports of neurodevelopmental outcomes in cohorts of children. Given the high prevalence of transient disease in the population of children with CHI we sought to assess neurodevelopment outcomes and compare these with data from patients with P-CHI.

## Aims

1. To test the hypothesis that the early childhood incidence of abnormal neurodevelopment is greater in children with P-CHI than in T-CHI.
2. To investigate factors associated with an increased risk of abnormal neurodevelopment in patients with T-CHI and P-CHI.

## Methods

The clinical notes of a cohort of children with CHI (*n* = 67), were examined retrospectively to assess abnormal neurodevelopment. The diagnosis of CHI was made using established criteria (Arnoux et al., 2012; Banerjee et al., 2011). All children had received diazoxide as first line treatment of CHI. Patients with perinatal asphyxia, prematurity <32 weeks or intraventricular hemorrhages were excluded from the study. Only those children within the age range 2.5–5 years were chosen for review. The lower age limit ensured that the child was old enough for abnormal neurodevelopmental outcomes to be manifest in routine clinical review. Childhood development was assessed at 6 months after the diagnosis of CHI, initially by a pediatric endocrinologist at our CHI Treatment. If developmental assessment was initially reported as normal, the child’s most recent appointment at a multidisciplinary CHI clinic was reviewed for evidence of possible abnormal neurodevelopment. If abnormal, a referral was made to a local community pediatrician for a formal developmental assessment. These assessments were analyzed retrospectively to derive a summated score of mild or severe abnormal neurodevelopment, based on observations in the following developmental domains: speech and language, motor, and vision (Meissner et al., 2003). While mild abnormal neurodevelopment was indicated by the presence of any neurological deficit or developmental delay, not within normal limits, severe abnormal neurodevelopment was indicated by the presence of significant functional impairment requiring sustained supportive therapy at home and at school.

Note was made if any child had had recurrent seizures beyond the first week after diagnosis, and if lower limb weakness was present (unilateral or bilateral). If infantile spasms were suspected, they were confirmed by the finding of hypsarrhythmia patterns in electroencephalography. Where available, brain magnetic resonance (MR) imaging results were noted. The long-term follow up of children with abnormal neurodevelopment was not recorded for this study. Formal psychometric testing was not performed in these children as this method of testing is reserved for those children older than 6 years in our Center.

All children were tested for *ABCC8*/*KCNJ11* mutations and other CHI-causing genes. All children who underwent surgery were diagnosed with focal or diffuse disease. Maximal dose of diazoxide (mg/kg/day) was recorded as a surrogate marker of severity of CHI, with greater dosage indicating a more severe disease (Banerjee et al., 2011). In addition, treatment with glucagon infusion to achieve glycemic stability and carbohydrate requirement (mg/kg/min) were recorded as severity markers, although the latter was recorded only in those with neonatal presentation, and not in those with late-presenting CHI.

Persistent congenital hyperinsulinism was defined in those children who remain on diazoxide or other medication or required pancreatectomy (focal or subtotal) to treat hypoglycemia. T-CHI was defined in those cases where hypoglycemia has resolved spontaneously and all medications have been discontinued to date (Banerjee et al., 2011).

Chi square and Fisher’s exact tests were used to test the difference between proportions, such as the incidence of abnormal neurodevelopment in P-CHI and T-CHI. Backward logistic regression models were used to test the association of covariates on binary outcomes, such as developmental outcome which was classified as either abnormal neurodevelopment or normal neurodevelopment.

## Results

In the whole cohort (*n* = 67), the median (range) age at diagnosis was 2 (1; 630) days, with insulin levels of 18.2 (2.1; 335.9) mU/l and glucose levels of 1.6 (0.1; 2.6) mmol/l. Maximum dose of diazoxide was 8.6 (4.5; 25.0) mg/kg/day, while glucagon infusion for initial glycemic stabilization was required in 13 (19%) children. In those with neonatal presentation, carbohydrate requirement was 18 (8; 25) mg/kg/min. There was a preponderance of males (*n* = 48, 72%) in the cohort. Birth weight was variable at 3.3 (1.2; 5.5) kg, with prematurity (32–37 weeks gestation) present in 13 (19%). The total number of CHI related mutations was 22 (33%), of which the majority were K_ATP_ channel gene mutations (*n* = 19). Other mutations included one each for *GCK*, *HADH*, and *PHOX2B*.

### CHI outcomes: T-CHI and P-CHI

Focal lesionectomy was performed in four children with focal CHI (6% of the cohort), and subtotal pancreatectomy was required for the treatment of medically unresponsive diffuse CHI in eight children (12% of the cohort). Spontaneous resolution of hypoglycemia occurred in children with T-CHI (*n* = 33, 49%), at a median (range) time of 119 (14; 700) days, while symptoms were sustained in P-CHI (*n* = 34, 51%) at a follow-up of 1187 (270; 4678) days. Clinically relevant diagnostic and treatment characteristics of children with T-CHI and P-CHI are given in Table 1. Early onset of CHI, i.e., age at presentation <7 days was more frequent in those with T-CHI. As expected, the presence of genetic mutations and the maximum dose of diazoxide were lower in those with T-CHI.

**Table 1:** **Diagnostic and treatment characteristics of children with T-CHI and P-CHI: categorical variables are represented as numbers of patients and as a percentage (%) of the cohort, while continuous variables are represented as median (range) values**.

	T-CHI (*n* = 33)	P-CHI (*n* = 34)	*p* Value for difference
Presentation <7 days, *n* (%)	30 (91%)	21 (62%)	0.009
Serum insulin at diagnosis (mU/l)	10.6 (2.1; 335.9)	18.2 (3; 132)	0.16
Serum glucose at diagnosis (mmol/l)	1.4 (0.1; 2.6)	1.6 (0.2; 2.6)	0.59
CHI-causing mutations, *n* (%)	4 (12%)	18 (53%)	<0.001
Carbohydrate requirement (mg/kg/min)	14 (8; 18.7)	18 (12.7; 21.0)	0.20
Numbers of patients requiring glucagon infusion, *n* (%)	6 (18%)	7 (20%)	0.80
Maximum dose of diazoxide requirement (mg/kg/day)	6.0 (5; 15)	15 (5; 25)	<0.001

### Normal and abnormal neurodevelopmental outcomes

Abnormal neurodevelopment was noted in 26 (39%) children in the whole cohort, with 8 (12%) children being mild and 18 (27%) children in the severe category. Within the abnormal neurodevelopment group, the prevalence of speech and language, motor and vision abnormalities, seizures, infantile spasms, and lower limb weakness is described in Table 2. Visual abnormalities, lower limb weakness, and infantile spasms occurred more frequently in those with severe abnormal neurodevelopment. The prevalence of severe abnormal neurodevelopment was similar in those presenting <7 or >7 days of age [16 (70%) vs. 2 (67%), *p* = 0.9].

**Table 2:** **Mild and severe abnormal neurodevelopment characterized in the domains of speech and language, motor, vision, seizures, infantile spasms, and limb weakness**.

	Mild abnormal neurodevelopment *n* = 8 children	Severe abnormal neurodevelopment *n* = 18 children	*p* Value for difference
Speech and language, *n* (%)	7 (87)	18 (100)	0.12
Motor, *n* (%)	6 (75)	18 (100)	0.08
Vision, *n* (%)	0 (0)	10 (56)	0.009
Seizures, *n* (%)	3 (37)	9 (50)	0.68
Infantile spasms, *n* (%)	0 (0)	4 (29)	0.26
Lower limb weakness, *n* (%)	0 (0)	8 (44)	0.03

**Figure 1:**
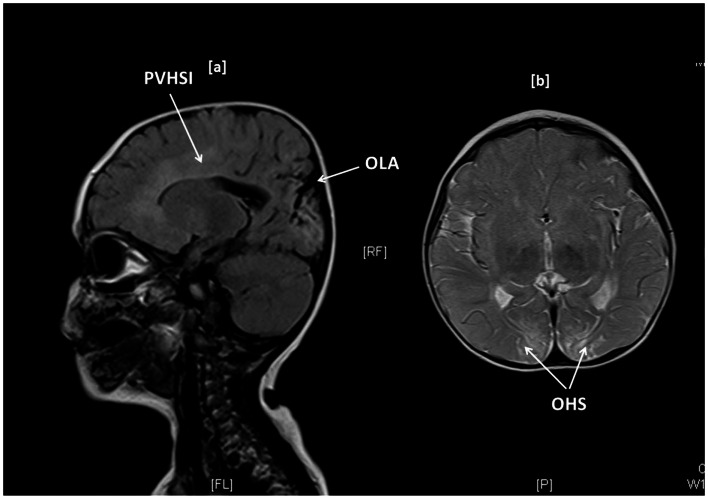
**Brain MR imaging in a 1-year-old girl with transient CHI, showing occipital lobe atrophy (OLA) and periventricular high signal intensities (PVHSI) in sagittal T2 FLAIR sequence scanning (1a) and occipital hyperintense signals (OHS) within subcortical white matter in proton density and T2 axial images, the latter being highly suggestive of hypoglycemic injury**.

Brain MR scanning was performed in 15 children with abnormal motor neurodevelopment, severe cognitive dysfunction, and seizures. Significant MR changes such as parietal infarcts, occipital high signal intensities, gliosis, infarctions, periventricular leukomalacia, basal ganglia changes, white matter atrophy, and sagittal sinus thrombosis were noted in 11 (73%) children. Such changes were present in children with T-CHI and P-CHI with similar frequencies [7 (87%) vs. 4 (57%), *p* = 0.18] but with a predictably greater frequency in those with severe abnormal neurodevelopment [10 (91%) vs. 1 (25%), *p* = 0.03]. The MR scan of one child with T-CHI with severe abnormal neurodevelopment outcome is shown in Figure 1. Two children with P-CHI, one with a focal lesion due to paternal *ABCC8* mutation, and the other with a paternally inherited *GCK* mutation, developed autism, but did not have MR changes. Severe abnormal neurodevelopment was not observed in siblings of children with CHI, negating the possibility of familial neurodevelopmental disorders.

**Figure 2:**
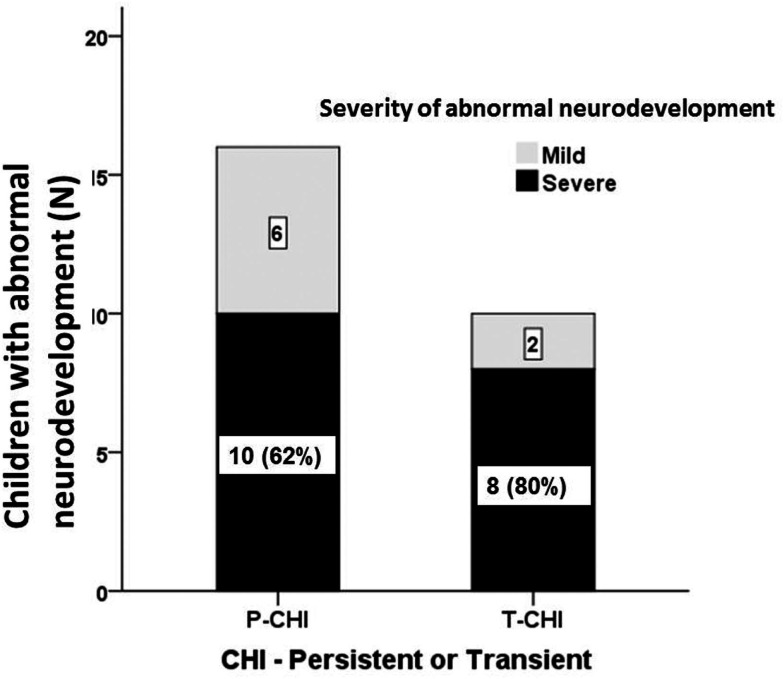
**The severity of abnormal neurodevelopment in patients with CHI**. Mild and severe abnormal neurodevelopment for both persistent (P-CHI) and transient CHI (T-CHI); the prevalence of severe abnormal neurodevelopment is similar in both groups, implying that early severity, but not the duration of hypoglycemia is important in determining the outcome of hypoglycemia in children with CHI.

The proportion of children with abnormal neurodevelopment in T-CHI was not significantly different from those in P-CHI [10 (30%) vs. 16 (47%), *p* = 0.15]. Further, the prevalence of severe abnormal neurodevelopment in patients with T-CHI was similar in those with P-CHI [8 (73%) vs. 10 (62%), *p* = 0.69] (Figure 2). These results refute our initial hypothesis that abnormal neurodevelopment occurs more commonly in P-CHI than T-CHI.

Diagnostic and treatment characteristics of children with normal and abnormal neurodevelopment are shown in Table 3. Presentation <7 days was more frequent in children with abnormal neurodevelopment. In children with neonatal presentation of CHI, carbohydrate requirement was similar in those with normal and abnormal neurodevelopment [18.0 (11.7; 25.0) vs. 15 (10.0; 25.0) mg/kg/min, *p* = 0.53]. The maximum dose of diazoxide, but not prematurity, genotype, carbohydrate requirement, or requirement for glucagon infusion was associated with abnormal neurodevelopment. In backward stepwise logistic regression (*R*^2^ = 0.26, *p* = 0.01), maximum dose of diazoxide [Odds ratio (95% confidence intervals) 1.3 (1.1; 1.5), *p* = 0.03] and age at presentation <7 days [5.9 (1.3; 27.8), *p* = 0.02], but not other covariates, such as gender, birth weight, prematurity, genotype, glucagon use, or the incidence of focal CHI were positively correlated with abnormal neurodevelopment.

**Table 3:** **Diagnostic and treatment characteristics in children with normal and abnormal neurodevelopment: categorical variables are represented as numbers of patient and as a percentage (%) of the cohort, while continuous variables are represented as median (range)**.

	Normal neurodevelopment *n* = 41 children	Abnormal neurodevelopment *n* = 26 children	*p* Value for difference
Presentation <7 days, *n* (%)	28 (68)	23 (89)	0.05
Insulin at diagnosis (mU/l)	13.4 (2.1; 335.9)	15.1 (3.2; 92)	0.52
Glucose at diagnosis (mmol/l)	1.6 (0.1; 2.6)	1.5 (0.2; 2.5)	0.61
CHI-causing mutations, *n* (%)	13 (32)	9 (35)	0.80
Carbohydrate requirement (mg/kg/min)	18 (8; 25)	15 (10; 25)	0.65
Number of patients requiring glucagon infusion, *n* (%)	7 (17%)	6 (23)	0.54
Maximum dose of diazoxide (mg/kg/day)	6.6 (2.0; 15.0)	10.0 (5.0; 25.0)	0.05
Focal CHI, *n* (%)	1 (2)	3 (11)	0.12
Time to spontaneous resolution (T-CHI), days	119 (14; 700)	163 (90; 699)	0.09

## Discussion

While abnormal neurodevelopment has been reported in significant proportions of children with P-CHI (Menni et al., 2001; Meissner et al., 2003), there are no reports of the incidence of abnormal neurodevelopment in T-CHI. Here, we report for the first time that a significant proportion of children with transient disease – 30% in our cohort, have abnormal neurodevelopment, often severe and associated with the presence of seizures. Therefore, although T-CHI may be perceived as a relatively “mild” illness, the long-term impact of neuroglycopenia causing adverse neurodevelopment may be significant and under-diagnosed.

It has been suggested that early onset hypoglycemia due to disorders such as CHI may be responsible for later onset epilepsy in childhood, even if transient in duration (Ng, 2012) and our findings add further support to this possibility. There is accumulating evidence that the neonatal brain may be susceptible to injury at a time when CHI usually manifests. In a recent study, although not in patients with CHI, hypoglycemia in the first 24 h after birth increased the probability of corticospinal tract damage 3.7-fold in neonates at risk of encephalopathy (Tam et al., 2012). Further, it has been noted that the presence of co-morbidities increases the likelihood of abnormal neurodevelopment in several disorders of hypoglycemia, including CHI (Gataullina et al., 2012). It is likely that neuroglycopenia in the early neonatal period may be a significant risk factor for abnormal neurodevelopment and that the first few weeks of life may represent an opportunity for aggressive correction of hypoglycemia to improve long-term outcomes in children with CHI, particularly as there is emerging evidence that the topography of brain injury is determined by the age of hypoglycemic insult (Gataullina et al., 2013). This therapeutic opportunity applies to children with both T-CHI and P-CHI and may apply to other conditions with neonatal hypoglycemia.

The overall incidence of abnormal neurodevelopment in children with P-CHI is similar to other studies over the last decade (Menni et al., 2001; Meissner et al., 2003; Ludwig et al., 2011), suggesting that despite major advances in the diagnosis and treatment of CHI, adverse neurodevelopmental outcome has not improved significantly. Although it has been suggested that satisfactory long-term neurological outcomes can be achieved in some populations of patients treated conservatively without pancreatectomy (Mazor-Aronovitch et al., 2007), on closer scrutiny of this study involving relatively small patient numbers (*n* = 21), 38% children required support for developmental delay in early life and 29% had learning problems later in school, implying that abnormal neurodevelopment rates were similar to other larger studies.

In our cohort the association of abnormal neurodevelopment with an early neonatal presentation is consistent with the report of Menni et al. (2001), but contradicts the findings of another study in 114 patients, which indicated that the correlation of abnormal neurodevelopment in P-CHI occurred with *infancy*-onset disease (Meissner et al., 2003). The difference could be explained by a bias in patient selection, delayed diagnosis in the infantile-onset group and the inherent variability in the retrospective analyses of questionnaires which were used to assess psychomotor development. It may be noted that there was no difference in the incidence of severe abnormal neurodevelopment presenting before or after the age of 7 days in our cohort.

In keeping with a case series (Kumaran et al., 2010), we found a disproportionate representation of children with infantile spasms in our cohort. It is possible that early onset hypoglycemia is a risk factor for the later development of hypsarrhythmias, although it is possible that common factors underpin the development of CHI and neuronal abnormalities. Studies in larger cohorts are required to confirm the association.

Our findings have weaknesses inherent to any observational study. The design was retrospective, the developmental assessments were not standardized and performed opportunistically at any time 6 months after diagnosis of CHI. However, the fact that the prevalence of neurological complications is broadly in keeping with other studies implies that the presence of abnormal neurodevelopment was appropriately detected in our study. It is possible that the study protocol under-reported the presence of mild abnormal neurodevelopment. However, it is unlikely that severe cases of abnormal neurodevelopment were missed, as all cases with normal neurodevelopment were subsequently reviewed by a clinical psychologist. Ideally, a prospective, formal cognitive, and psychomotor testing protocol in all patients from diagnosis would be preferred. However, this is likely to be resource intensive and time consuming, in contrast to our retrospective and observational study protocol. Further, it might have been difficult to capture abnormal neurodevelopment in T-CHI patients, as some children achieved spontaneous resolution late. It is well recognized that T-CHI may resolve later, with reports of resolution up to 8 years of age (Banerjee et al., 2011); indeed, the very late resolution of hypoglycemia also implies that some children with P-CHI at this time may possibly be categorized as T-CHI at a later date.

In our study, brain MR scanning was not performed routinely; instead scans were requested on the basis of clinical need. In those with abnormal neurodevelopment, MR scan was informative, but not diagnostic of the abnormal neurodevelopment. Further, in some children, MR scanning did not show anatomical abnormalities, suggesting that brain imaging may be complimentary but not independently useful in the neurodevelopmental assessment of children with CHI, although recent evidence suggests that the timing of hypoglycemic injury may influence the anatomical distribution of brain damage (Gataullina et al., 2013).

The correlation of abnormal neurodevelopment with diazoxide dose as a marker of severity is interesting and novel. Although maximum carbohydrate requirement at diagnosis might be the rational marker of choice denoting severity of hypoglycemia, this measurement cannot always be determined, as supported by evidence from other studies (Meissner et al., 2003). This is because the glucose content of food in the post-weaning period cannot be reliably calculated, unless nutrition is prescribed for gastrostomy feeding or the child is on intravenous fluids. Although we did record carbohydrate intake, we used escalating doses of diazoxide as a surrogate marker of insensitivity to the drug and therefore of the severity of CHI. Theoretically, it is possible that the positive correlation of diazoxide dose and abnormal neurodevelopment indicates that diazoxide itself may be detrimental to the neonatal brain. However, there have been no clinical reports of brain injury from diazoxide use, and there are several studies in animals demonstrating neuroprotective effects of diazoxide (Zhang et al., 2010, Huang et al., 2010). It is possible that diazoxide unresponsiveness represents an independent adverse marker for abnormal neurodevelopment, due to relative drug inactivity at the level of neuronal K_ATP_ channels, which are important for brain neuronal survival. Further work in animal models and correlation in human beings is required to evaluate the independent role of brain K_ATP_ channels in the pathogenesis of abnormal neurodevelopment.

## Conclusion

Abnormalities in childhood development are common in children with transient forms of CHI, with about a third being affected. This rate is similar to that in children with persistent forms of the disease. Severe and early onset hypoglycemia may adversely influence later neurodevelopmental outcomes. Therefore, in children with CHI, early recognition of hypoglycemia, followed by rapid correction may improve the morbidity of learning and physical disabilities in later life.

## Conflict of Interest Statement

The authors declare that the research was conducted in the absence of any commercial or financial relationships that could be construed as a potential conflict of interest.

## References

[B1] Arnoux J. B. Verkarre V. Saint-Martin C. Montravers F. Brassier A. Valayannopoulos V. (2012). Congenital hyperinsulinism: current trends in diagnosis and therapy. Orphanet. J. Rare Dis. 6, 63 10.1186/1750-1172-6-63 21967988 PMC3199232

[B2] Bahi-Buisson N. Roze E. Dionisi C. Escande F. Valayannopoulos V. Feillet F. (2008). Neurological aspects of hyperinsulinism-hyperammonemia syndrome. Dev. Med. Child Neurol. 50, 945–949 10.1111/j.1469-8749.2008.03114.x 19046187

[B3] Banerjee I. Skae M. Flanagan S. E. Rigby L. Patel L. Didi M. (2011). The contribution of rapid KATP channel gene mutation analysis to the clinical management of children with congenital hyperinsulinism. Eur. J. Endocrinol. 164, 733–740 10.1530/EJE-10-1136 21378087

[B4] Gataullina S. Dellatolas G. Perdry H. Robert J. J. Valayannopoulos V. Touati G. (2012). Comorbidity and metabolic context are crucial factors determining neurological sequelae of hypoglycaemia. Dev. Med. Child Neurol. 54, 1012–1017 10.1111/j.1469-8749.2012.04400.x 22924392

[B5] Gataullina S. Lonlay P. D. Dellatolas G. Valayannapoulos V. Napuri S. Damaj L. (2013). Topography of brain damage in metabolic hypoglycaemia is determined by age at which hypoglycaemia occurred. Dev. Med. Child Neurol. 55, 162–166 10.1111/dmcn.12045 23205874

[B6] Gloyn A. L. Diatloff-Zito C. Edghill E. L. Bellanne-Chantelot C. Nivot S. Coutant R. (2006). KCNJ11 activating mutations are associated with developmental delay, epilepsy and neonatal diabetes syndrome and other neurological features. Eur. J. Hum. Genet. 14, 824–830 10.1038/sj.ejhg.5201629 16670688

[B7] Huang C. W. Wu S. N. Cheng J. T. Tsai J. J. Huang C. C. (2010). Diazoxide reduces status epilepticus neuron damage in diabetes. Neurotox. Res. 17, 305–316 10.1007/s12640-009-9104-3 19728004

[B8] Kapoor R. R. Flanagan S. E. Fulton P. Chakrapani A. Chadefaux B. Ben-Omran T. (2009). Hyperinsulinism-hyperammonemia syndrome: novel mutations in the GLUD1 gene and genotype-phenotype correlations. Eur. J. Endocrinol. 161, 731–735 10.1530/EJE-09-0615 19690084 PMC2761810

[B9] Kumaran A. Kar S. Kapoor R. R. Hussain K. (2010). The clinical problem of hyperinsulinemic hypoglycemia and resultant infantile spasms. Pediatrics 126, e1231–6 10.1542/peds.2009-2775 20956428

[B10] Ludwig A. Ziegenhorn K. Empting S. Meissner T. Marquard J. Holl R. (2011). Glucose metabolism and neurological outcome in congenital hyperinsulinism. Semin. Pediatr. Surg. 20, 45–49 10.1053/j.sempedsurg.2010.10.005 21186004

[B11] Mazor-Aronovitch K. Gillis D. Lobel D. Hirsch H. J. Pinhas-Hamiel O. Modan-Moses D. (2007). Long-term neurodevelopmental outcome in conservatively treated congenital hyperinsulinism. Eur. J. Endocrinol. 157, 491–497 10.1530/EJE-07-0445 17893264

[B12] Meissner T. Wendel U. Burgard P. Schaetzle S. Mayatepek E. (2003). Long-term follow-up of 114 patients with congenital hyperinsulinism. Eur. J. Endocrinol. 149, 43–51 10.1530/eje.0.1490043 12824865

[B13] Menni F. de Lonlay P. Sevin C. Touati G. Peigne C. Barbier V. (2001). Neurologic outcomes of 90 neonates and infants with persistent hyperinsulinemic hypoglycemia. Pediatrics 107, 476–479 10.1542/peds.107.3.476 11230585

[B14] Ng Y. T. (2012). The hitchhiker’s guide to the role of (transient) hypoglycemia in refractory seizures and epilepsy. Pediatr. Neurol. 47, 123–124 10.1016/j.pediatrneurol.2012.04.020 22759689

[B15] Senniappan S. Shanti B. James C. Hussain K. (2012). Hyperinsulinaemic hypoglycaemia: genetic mechanisms, diagnosis and management. J. Inherit. Metab. Dis. 35, 589–601 10.1007/s10545-011-9441-2 22231386

[B16] Tam E. W. Haeusslein L. A. Bonifacio S. L. Glass H. C. Rogers E. E. Jeremy R. J. (2012). Hypoglycemia is associated with increased risk for brain injury and adverse neurodevelopmental outcome in neonates at risk for encephalopathy. J. Pediatr. 161, 88–93 10.1016/j.jpeds.2011.12.047 22306045 PMC3346850

[B17] Vercellino G. F. Cremonte M. Carlando G. Colivicchi M. Crivelli S. Ricotti A. (2011). Transient neonatal hyperinsulinemic hypoglycemia and neurological outcome: a case report. Minerva Pediatr. 63, 111–114 21487374

[B18] Zhang H. Zhao D. Wang Z. Zheng D. (2010). Diazoxide preconditioning alleviates caspase-dependent and caspase-independent apoptosis induced by anoxia-reoxygenation of PC12 cells. J. Biochem. 148, 413–421 20616381 10.1093/jb/mvq074

